# Essential role for the TRF2 telomere protein in adult skin homeostasis

**DOI:** 10.1111/acel.12221

**Published:** 2014-04-14

**Authors:** Paula Martínez, Iole Ferrara-Romeo, Juana M Flores, Maria A Blasco

**Affiliations:** 1 Telomeres and Telomerase Group, Molecular Oncology Program, Spanish National Cancer Centre (CNIO)Melchor Fernández Almagro 3, E-28029, Madrid, Spain; 2 Animal Surgery and Medicine Department, Facultad de Veterinaria, Complutense University of Madrid28029, Madrid, Spain

**Keywords:** 53BP1, DNA damage, p53, skin embryonic development, Telomeres, TRF2

## Abstract

TRF2 is a component of shelterin, the protein complex that protects the ends of mammalian chromosomes. TRF2 is essential for telomere capping owing to its roles in suppressing an ATM-dependent DNA damage response (DDR) at chromosome ends and inhibiting end-to-end chromosome fusions. Mice deficient for TRF2 are early embryonic lethal. However, the role of TRF2 in later stages of development and in the adult organism remains largely unaddressed, with the exception of liver, where TRF2 was found to be dispensable for maintaining tissue function. Here, we study the impact of *TRF2* conditional deletion in stratified epithelia by generating the *TRF2*^*∆/∆*^*-K5-Cre* mouse model, which targets *TRF2* deletion to the skin from embryonic day E11.5. In marked contrast to *TRF2* deletion in the liver, *TRF2*^*∆/∆*^*-K5-Cre* mice show lethality in utero reaching 100% lethality perinataly. At the molecular and cellular level, *TRF2* deletion provokes induction of an acute DDR at telomeres, leading to activation of p53 signaling pathways and to programed cell death since the time of Cre expression at E11.5. Unexpectedly, neither inhibition of the NHEJ pathway by abrogation of 53BP1 nor inhibition of DDR by p53 deficiency rescued these severe phenotypes. Instead, *TRF2* deletion provokes an extensive epidermal cell death accompanied by severe inflammation already at E16.5 embryos, which are independent of p53. These results are in contrast with conditional deletion of *TRF1* and *TPP1* in the skin, where p53 deficiency rescued the associated skin phenotypes, highlighting the comparatively more essential role of TRF2 in skin homeostasis.

## Introduction

The ends of linear chromosomes are formed by special heterochromatic structure, known as the telomere, which protects them from degradation and repair activities and, it is essential to ensure chromosomal stability (de Lange, 2005; Blasco, 2007; Palm & de Lange, 2008). Mammalian telomeric chromatin is composed of tandem repeats of the TTAGGG sequence bound by a specialized protein complex known as shelterin (de Lange, 2005; Palm & de Lange, 2008). The shelterin complex is composed of six core proteins, the telomeric repeat binding factor 1 and 2, (TRF1 and TRF2), the TRF1-interacting protein 2 (TIN2), protection of telomeres protein 1 (POT1), TPP1, (also known as ACD, TINT1, PTOP, or PIP1), and RAP1 (de Lange, 2005). TRF1, TRF2, and POT1 bind directly to telomeric DNA repeats, with TRF1 and TRF2 binding to telomeric double-stranded DNA and POT1 to the 3′-single-stranded G-overhang (Broccoli *et al*., 1997; Bianchi *et al*., 1999; Court *et al*., 2005; de Lange, 2005; Palm & de Lange, 2008). (Baumann & Cech, 2001; Loayza & De Lange, 2003; Lei *et al*., 2004; He *et al*., 2006; Hockemeyer *et al*., 2006; Wu *et al*., 2006; Palm & de Lange, 2008). TIN2 is able to bind TRF1 and TRF2 through independent domains and to recruit the TPP1-POT1 complex, constituting the bridge between the different shelterin components (Kim *et al*., 2004; Liu *et al*., 2004; Ye *et al*., 2004; Chen *et al*., 2008). TPP1 recruits POT1 to telomeres (Chen *et al*., 2007; Kibe *et al*., 2010). In addition, TPP1 has a crucial role in the recruitment of telomerase to chromosome ends (Xin *et al*., 2007; Tejera *et al*., 2010; Nandakumar *et al*., 2012; Sexton *et al*., 2012; Zhong *et al*., 2012; Zhang *et al*., 2013). Finally, RAP1 binds telomeric repeats through its interaction with TRF2 although it is dispensable for telomere capping (Martinez *et al*., 2010; Sfeir *et al*., 2010). Rap1 has also been shown to bind throughout chromosome arms where it regulates gene expression. (Martinez *et al*., 2010). Indeed, Rap1 has been recently demonstrated to protect from obesity through its role in regulating key metabolic pathways (Martinez *et al*., 2013; Yeung *et al*., 2013).

Abrogation of TRF1, TRF2, POT1b, TPP1, and TIN2 in mice results in early embryonic lethality (Karlseder *et al*., 2003; Chiang *et al*., 2004; Celli & de Lange, 2005; Hockemeyer *et al*., 2006; Lazzerini Denchi *et al*., 2006; Wu *et al*., 2006; Kibe *et al*., 2010). Conditional deletion of *TRF1* and *TPP1* in mouse stratified epithelia leads to perinatal lethality coincidental with skin hyperpigmentation and severe skin morphogenesis defects, including absence of mature hair follicles and sebaceous glands, which are concomitant with induction of telomere-originated DNA damage, activation of the p53/p21 and p16 pathways, and cell cycle arrest *in vivo* (Martinez *et al*., 2009; Tejera *et al*., 2010). Importantly, p53 deficiency rescues both the stem cell defects and skin hyperpigmentation, as well as mouse survival, in both mouse models, indicating that the severe skin defects associated with TRF1 and TPP1 abrogation are mediated by p53. Interestingly, long-lived *TRF1/p53* double null mice spontaneously develop invasive and genomically unstable squamous cell carcinomas (Martinez *et al*., 2009). These results suggest that TRF1 normally acts as a tumor suppressor in the context of the organism by preventing telomere-induced genetic instability in proliferating cells. In marked contrast to TRF1 and TPP1, targeted *Rap1* deletion in stratified epithelia in *Rap1*^*Δ/Δ*^ *K5-cre* mice does not impact on mouse viability in accordance with a dispensable role for Rap1 in telomere capping (Martinez *et al*., 2010).

In contrast to conditional deletion of *TPP1* and *TRF1* in stratified epithelia, *TRF2* conditional deletion in the liver (*Mx1TRF2* mice) did not impact on liver regeneration or mouse viability, indicating that TRF2 is dispensable for hepatocyte regeneration (Lazzerini Denchi *et al*., 2006). In particular, TRF2 deletion in hepatocytes leads to telomere damage and telomere fusions; however, these phenotypes were not accompanied by loss of liver function upon partial hepatectomy. Indeed, liver regeneration in these mice occurred by endoreduplication and cell growth, but not cell division, thereby overcoming the chromosome segregation problems associated with telomere fusions (Lazzerini Denchi *et al*., 2006).

To further understand the role of TRF2 in the adult organism, as well as to do a comparative analysis with conditional knock-out models for other shelterins, here we deleted *TRF2* in stratified epithelia. *TRF2*^*Δ/Δ*^*K5-Cre* mice were born at submendelian ratios indicating partial embryonic lethality. Newborn mice die immediately after birth and show complete lack of epidermis. We show that *TRF2*-deleted epidermis undergoes tissular necrosis accompanied by the presence of dermal infiltrates and increased IL6 levels already at E13.5 of embryonic development that becomes apparent at E16.5 by the complete absence of the epidermal layer of the skin. Comparative studies of the phenotypic effects originated by conditional deletion of either *TRF1*, *TPP1*, *RAP1,* or *TRF2* in stratified epithelia, a highly proliferative tissue, revealed that TRF2 deficiency causes the most severe proliferative and developmental defects of all these shelterin components.

## Results

### *TRF2* conditional deletion in stratified epithelia results in partial embryonic lethality and impairs skin development

**Figure 1:**
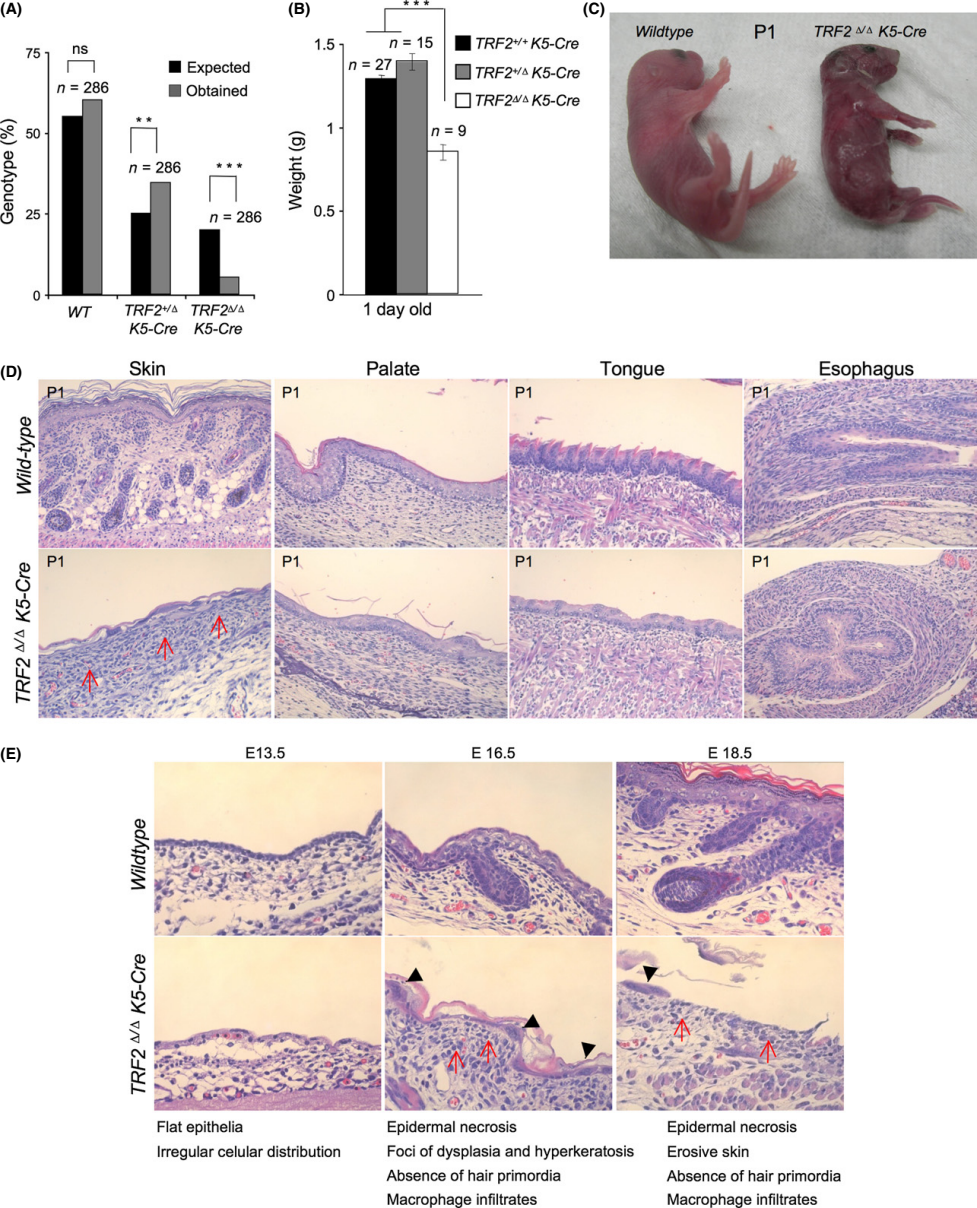
TRF2 deletion in stratified epithelia results in partial embryonic lethality and impedes skin embryonic development. (A) *TRF2*^*∆/∆*^ *K5-Cre* mice were born at submendelian ratios. *N*, number of mice. The χ^2^ test was used to determine statistical significance. (B) *TRF2*^*∆/∆*^ *K5-Cre* mice are born with low body weight. *N*, number of mice. The Student’s *t*-test was used for statistical analysis. **P < 0.01; ***P < 0.001. (C) Representative images of wild-type and *TRF2*^*∆/∆*^ *K5-Cre* newborns. Note the shiny appearance due to complete absence of epidermal cells in *TRF2*^*∆/∆*^ *K5-Cre* newborns. (D) Histopathological representative images of the skin, palate, tongue, and esophagus. Note that necrosis of epidermal layer is evident as observed by complete absence of keratinocytes, dermal infiltrates, and dysplastic skin foci with hyperkeratosis. Red arrows mark macrophage and neutrophil dermal infiltrates. (E) Histopathological findings of the developing *TRF2*^*∆/∆*^ *K5-Cre* epidermis at stage E13.5, E16.5, and E18,5 of embryonic development. From E16.5, epidermal necrosis becomes evident. Black arrow heads mark foci of dysplasia and hyperkeratosis and red arrows mark macrophage and neutrophil dermal infiltrates.

*TRF2* conditional deletion in the liver was previously found to be dispensable for liver regeneration and mouse viability (Lazzerini Denchi *et al*., 2006). Here, we set to address *in vivo* the impact of TRF2 deficiency in a highly proliferative tissue, such as stratified epithelia. To this end, we crossed conditional *TRF2*^*flox/flox*^ mice or *TRF2*^*+/flox*^ mice with K5-Cre mice that constitutively express the Cre recombinase under the control of the keratin 5 (K5) promoter from day 11.5 of embryonic development onwards (Ramirez *et al*., 1994), thereby generating *TRF2*^*Δ/Δ*^*K5-Cre* mice. *TRF2*^*Δ/Δ*^*K5-Cre* mice were born at submendelian ratios indicating partial embryonic lethality associated with TRF2 abrogation in stratified epithelia (Fig. 1A). In addition, newborn *TRF2*^*Δ/Δ*^*K5-Cre* mice presented a lower body weight than their wild-type counterparts and died immediately after birth (Fig. 1B). At the macroscopic level, newborn *TRF2*^*∆/∆*^*-K5-Cre* mice showed a strongly compromised skin phenotype, with a very thin and fragile epidermis at the time of birth (Fig. 1C). In addition, histopathological analysis of newborn mice (P1) revealed severe abnormalities in the skin, palate, tongue, and esophagus. In particular, the skin of TRF2 deficient newborns showed rare small pieces of skin that were mostly necrotic and were devoid of hair follicles and sebaceous glands. Subjacent areas of dermal inflammation showed the presence of macrophages and neutrophils (red arrows in Fig 1D). *TRF2*^*∆/∆*^*K5-Cre* newborns also showed severely dysplastic epithelia in the tongue and palate, showing areas of atrophy and a reduced number of cellular layers, a phenotype that was milder in the esophagus (Fig. 1D). Of note, no significant phenotype was observed in stratified epithelia of heterozygous *TRF2*^*+/∆*^*-K5-Cre* mice compared with the control *TRF2*^*+/+*^ mice (data not shown).

Given the very severe skin defects of TRF2 deficient mice at the time of birth, we set to analyze the impact of *TRF2* deletion at earlier stages of skin embryonic development. To this end, we studied embryos at embryonic stages E11.5 (time of induction of *TRF2* deletion), E13.5, E16.5, and E18.5 (Fig. 1E and Fig. S1). From E11.5, a progressive reduction in the skin cellularity was observed both in the suprabasal and in the basal skin layers (Fig. S1). At day E13.5, the *TRF2*^*∆/∆*^*-K5-Cre* epidermis presented a flat epithelia containing just one keratinocyte layer with irregular cellular distribution compared with the two keratinocyte layers present in the wild-type controls (Fig. 1E). At E16.5, wild-type skin presented four keratinocyte layers, while the *TRF2*^*∆/∆*^*-K5-Cre* epidermis was largely necrotic as observed by almost complete absence of the basal cell layer and showing dysplastic skin foci with hyperkeratosis (Fig. 1E). Diffuse dermal inflammation is evident (red arrows in Fig. 1E). At this stage, the skin lacked early hair primordial and was composed of only one layer of keratinocytes showing atypically large nuclei (black arrow heads in Fig. 1E). In addition, suprabasal layer development and differentiation were completely blocked in the absence of TRF2. These skin defects progressively worsened until E18.5 at which point almost no keratinocytes could be detected except for a few skin patches with aberrantly large nuclei, suggestive of endoreduplication and massive necrosis of the tissue (black arrow heads in Fig. 1E; see Fig. 4). These rare skin patches also showed complete absence of hair primordia (Fig. 1E). From E18.5 onwards, TRF2-null skin was erosive and composed of a very thin stratum corneum completely lacking hair follicles and sebaceous glands (Fig. S1).

Immunohistochemical analysis of various epidermal differentiation markers, including cytokeratin 10, cytokeratin 14, cytokeratin 6, and loricrin, at different stages of embryonic development revealed an abnormal expression pattern in the absence of TRF2 (Fig. S1), confirming a strongly compromised skin development from day E11.5 when K5-Cre begins to be expressed (E11.5) (Ramirez *et al*., 1994). In particular, while loricrin and cytokeratins K10 and K14 showed a reduced expression, the cytokeratin 6 showed a higher expression and abnormal distribution compared wild-type skin (Fig. S1).

### Severe epidermal stem cell defects in TRF2^*∆/∆*^ K5-Cre mouse epidermis

Impairment of skin development in *TRF2*^*∆/∆*^*-K5-Cre* mice suggested a defect in the proliferative capacity of epidermal stem cells. We examined the impact of *TRF2* abrogation on epidermal stem cell functionality by studying the expression of p63, a marker of proliferating cells of the basal skin layer in stratified epithelia, and Sox9, a stem cell gene that establishes epidermal stem cell compartments in mice during embryonic and early postnatal development (Vidal *et al*., 2005; Nowak *et al*., 2008). p63 staining pattern revealed that TRF2-null skin already at E11.5 presents a strong reduction in the number of basal cells becoming completely absent at day E18.5 compared with wild-type skin (Fig. S2).

Sox9 is expressed early in skin morphogenesis and is essential to establish the stem cell niche, so that in its absence, the early hair bulge stem cell population never forms and hair bulge and sebaceous glands do not develop (Vidal *et al*., 2005; Nowak *et al*., 2008). Wild-type embryos showed an intense and widespread signal of Sox9 at E13.5, and starting on E16.5, the expression of Sox9 was restricted to the early hair primordia and to the hair bulge in the newborns. In contrast, Sox9 expression in *TRF2*-deleted epidermis was only detected at E13.5 although to a much lesser extend as compared to wild-type counterparts. Sox9 expression is undetectable from E16.5, in accordance with the complete absence of hair primordia (Fig. S2).

### *TRF2* deletion in epidermis leads to a persistent DDR activation

**Figure 2:**
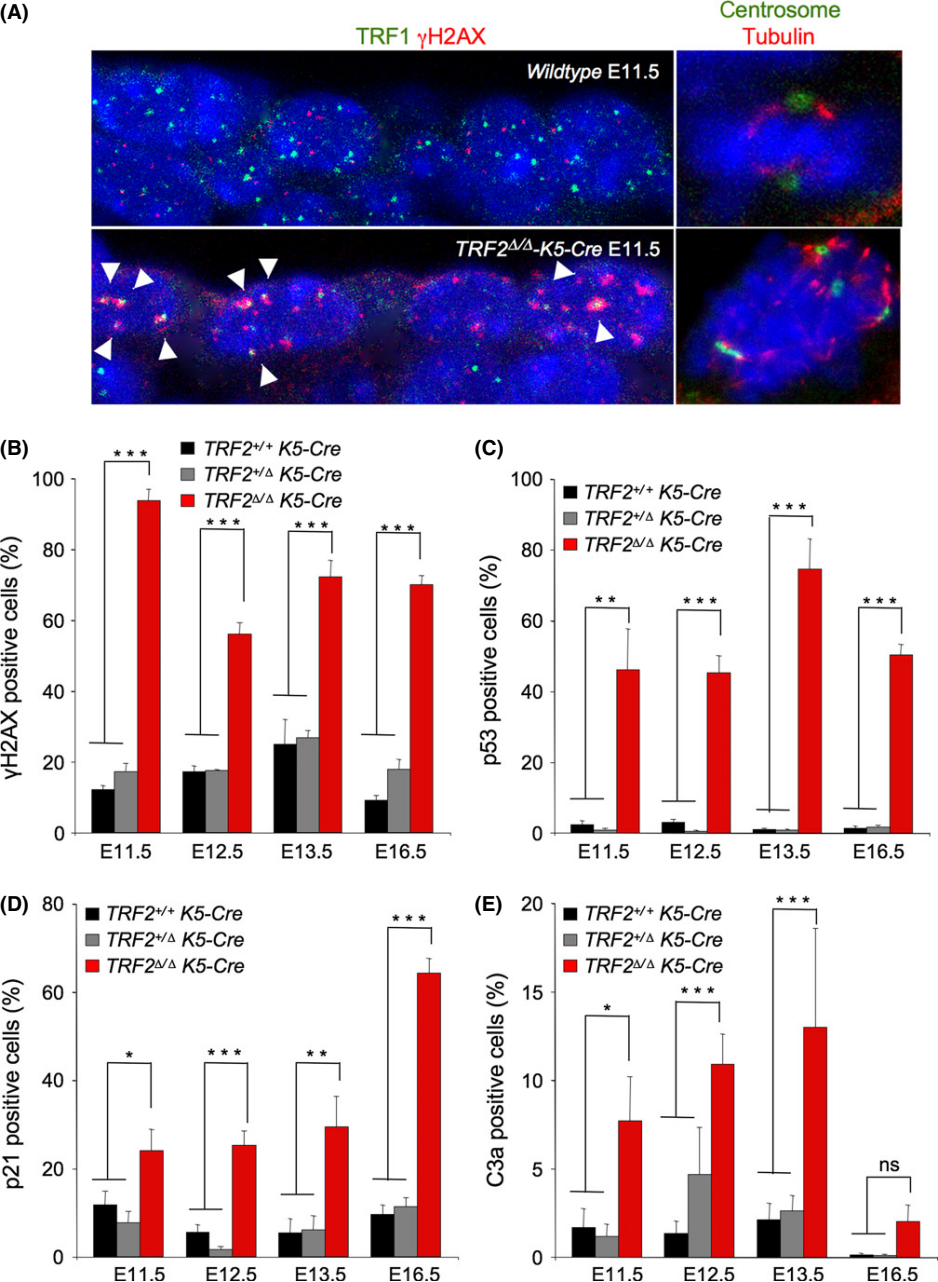
TRF2 deletion in mouse epidermis induces massive telomeric DNA damage and its associated DNA damage response. (A) Representative image of epidermal cell layer of E11.5 embryo sections of the indicated genotypes stained for TRF1 and γH2AX (left panel) and for pericentrin and α-tubulin (right panel). White arrows indicate colocalization of DNA damage foci to telomeres (TIFS). Note the presence of multipolar mitosis with more than two centrosomes observed in the absence of TRF2 indicative of fused chromosomes. (B–E) Percentage of epidermal cells showing γH2AX foci (B), p53 positive cells (C), p21 positive cells (D) and apoptotic C3a positive cells (E) in skin sections of wild-type, *TRF2*^*+/∆*^ *K5-Cre* and *TRF2*^*∆/∆*^ *K5-Cre* embryos at the indicated stages of embryonic development. *n* = 2–3 mice analyzed for genotype. Error bars indicate standard error. Student’s *t*-test was used for statistical analysis. *P = 0.05; **P < 0.01; ***P < 0.001.

*TRF2* deletion in MEFs is known to result in induction of a DNA damage response (DDR), genome-wide telomere fusions, and onset of p53/Rb-dependent cellular senescence (Celli & de Lange, 2005). Uncapped telomeres are recognized as DNA double-strand breaks (DSB) and, as such, are bound by 53BP1 and γH2AX forming the so-called telomere-induced foci (TIF) (Takai *et al*., 2003). To address the presence of DNA damage specifically at telomeres in the absence of TRF2, we made a double immunofluorescence staining with anti-TRF1 and anti-γH2AX in E11.5 embryos. We find that epidermal cells lacking TRF2 show γH2AX foci and that the majority of γH2AX foci colocalize with TRF1 foci in *TRF2*^*∆/∆*^*-K5-Cre* embryos but not in wild-type embryos indicating that *TRF2* deletion in stratified epithelia resulted in a rapid induction of DNA damage specifically at telomeres (Fig. 2A, left panel: see below quantification of γH2AX-positive cells in the different genotypes). In addition, *TRF2*-deleted keratinocytes showed multipolar mitosis containing more than two centrosomes indicative of fused chromosomes in the absence of TRF2 (Fig. 2A, right panel).

To address whether complete impairment of skin development in *TRF2*^*∆/∆*^ *K5-Cre* mice was also associated with induction of DNA damage, we quantified the abundance of γH2AX positive cells during skin development using immunohistochemistry (Experimental Procedures). Given the almost complete absence of keratinocytes in TRF2-null skin at day E18.5, we focused our analysis in E11.5, E12.5, E13.5, and E16.5 wild-type, *TRF2*^*+/∆*^ heterozygous and *TRF2*^*∆/∆*^ deficient embryos (Fig. 2). At all the analyzed time points, we observed an increased number of γH2AX-positive cells in *TRF2*^*∆/∆*^*-K5-Cre* epidermis compared with wild-type epidermis. At E11.5, upon initial deletion of the gene, around 90% of cells were γH2AX-positive as compared to approximately 15% observed in heterozygous and in wild-type controls (Fig 2B). The percentage of cells showing γH2AX foci decreased in *TRF2*-null skin sections in the following days, remaining stable at 75% at E13.5 and E16.5, while less than 10% were γH2AX-positive in the controls (Fig. 2B). This decrease in the number of γH2AX-positive cells could be due to a rapid induction of chromosome fusions upon *TRF2* deletion at E11.5 that consequently lowered the number of exposed uncapped γH2AX labeled telomeres (Fig. 2A, left panel).

Persistent telomere damage, such as that induced by telomere uncapping, leads to DNA damage response (DDR) characterized by activation of cell cycle inhibitors p53/p21 that leads to cessation of cellular proliferation (de Lange, 2009; Martinez & Blasco, 2010, 2011). Indeed, already at E11.5 *TRF2*^*∆/∆*^*-K5-Cre* embryos showed 15-fold and twofold increase in the amount of p53 and p21 positive cells, respectively, compared with heterozygous and wild-type embryos. The number of p53 and p21 positive cells gradually increased up to 60% at E16.5 in agreement with the severe proliferative defects observed in *TRF2*-deleted skin (Fig. 2C,D). Interestingly, TRF2 depletion was shown to initially induce cellular apoptosis. Thus, the number of active caspase-3 positive cells was progressively augmenting from E11.5 to E.13.5 in *TRF2*^*∆/∆*^*-K5-Cre* embryos, from approximately 8 to 15% as compared to 3% in the heterozygous and wild-type embryos (Fig. 2E). However, at E16.5, epidermal necrosis is evident as almost no basal cells are present in *TRF2*^*∆/∆*^*-K5-Cre* embryos (Fig. 1E). Indeed, not even apoptotic cells are observed at this stage (Fig. 2E). These results indicate that TRF2 deletion leads to a progressive loss of skin cellularity initially owing to induction of senescence and apoptosis and eventually resulting in complete skin loss due most likely to epidermal necrosis (see Fig. 4).

In accordance with normal skin development and mouse survival, *TRF2*^*+/Δ*^ heterozygous mice showed no significant differences in the number of γH2AX, p53, p21, and C3a expressing cells compared with wild-type embryos (Fig. 2B–E).

## 53BP1 deficiency does not rescue TRF2-associated developmental defects

**Figure 3:**
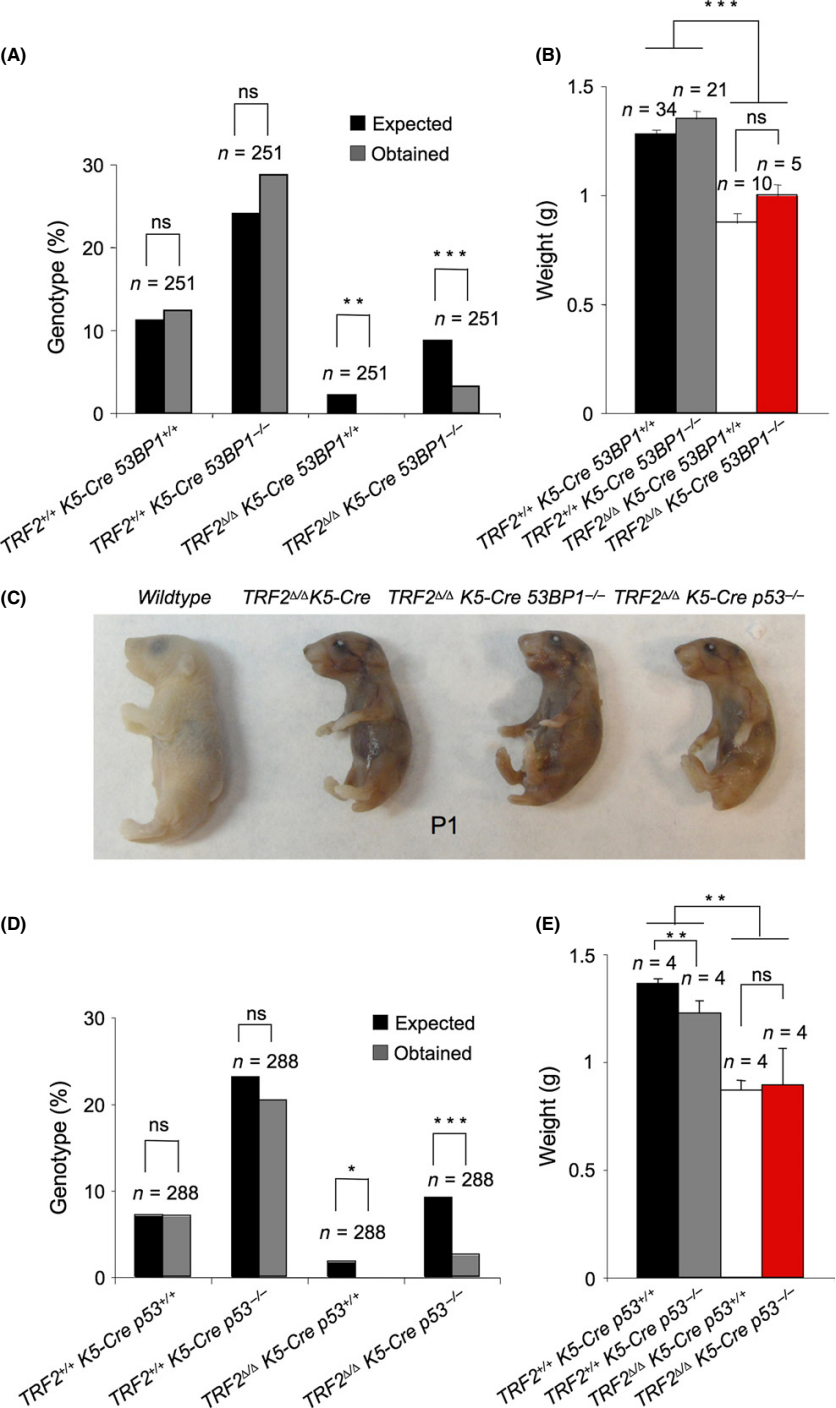
53BP1 and p53 deficiencies do not rescue TRF2-associated developmental defects. (A) *TRF2*^*∆/∆*^ *K5-Cre 53BP1*^*−/−*^ mice were born at submendelian ratios. *N*, number of mice. (B) *TRF2*^*∆/∆*^ *K5-Cre* and *TRF2*^*∆/∆*^ *K5-Cre 53BP1*^*−/−*^ mice do not differ in body weight at birth. (C) Representative images of wild-type, *TRF2*^*∆/∆*^ *K5-Cre, TRF2*^*∆/∆*^ *K5-Cre 53BP1*^*−/−*^ and *TRF2*^*∆/∆*^ *K5-Cre p53*^*−/−*^ newborns. (D) *TRF2*^*∆/∆*^ *K5-Cre p53*^*−/−*^ mice were born at submendelian ratios. (E) *TRF2*^*∆/∆*^ *K5-Cre* and *TRF2*^*∆/∆*^ *K5-Cre p53*^*−/−*^ mice do not differ in body weight at birth. *N*, number of mice. The χ^2^ test was used to determine statistical significance in a and d. The Student’s *t*-test was used for statistical analysis in b and e. *P = 0.05; **P < 0.01; ***P < 0.001.

53BP1 is a C-NHEJ component and an ATM target that accumu-lates at DSBs and at uncapped telomeres (Rappold *et al*., 2001; Fernandez-Capetillo *et al*., 2002; Wang *et al*., 2002; d’Adda di Fagagna *et al*., 2003; Takai *et al*., 2003). It has been shown that chromosome end-to-end fusions occurring upon *TRF2* deletion are strongly dependent on the binding of 53BP1 to damaged chromosome ends and that in the absence of 53BP1, these fusions can be rescued (Dimitrova *et al*., 2008; Rai *et al*., 2010). To address the contributions of aberrant chromosome rearrangements to the deleterious effects of *TRF2* deletion *in vivo*, we crossed conditional *TRF2*^*+/∆*^ *K5-Cre* with *TRF2*^*+/+*^ *53BP1*^*−/−*^ mice (Ward *et al*., 2003), to obtain *TRF2*^*∆/∆*^ *K5-Cre 53BP1*^*−/−*^ mice. Similar to *TRF2*^*Δ/Δ*^*K5-Cre* single knock-out mice (Fig. 1A,B), doubly deficient *TRF2*^*Δ/Δ*^*-K5-Cre 53BP1*^*−/−*^ mice present submendelian birth ratios, neonates had significantly lower body weight as compared to control mice and died immediately after birth (Fig. 3A–C). Neither differences in body weight nor macroscopic differences were revealed between *TRF2*^*Δ/Δ*^*K5-Cre* and *TRF2*^*Δ/Δ*^*K5-Cre 53BP1*^*−/−*^ neonates (Fig. 3B,C). These results indicate that abolishment of the C-NHEJ and consequently abolishment of chromosome fusions neither rescued nor aggravated the pathological effects of *TRF2* deletion in stratified epithelia (see Fig. 6D–E), suggesting that the presence of fusions is not responsible for the mouse phenotype.

### p53 deficiency does not rescue TRF2-associated developmental defects

TRF2 deficiency causes severe proliferative defects *in vitro* (Celli & de Lange, 2005). *TRF2*^*Δ/Δ*^*-K5-Cre* epidermis showed a strong induction of the cell cycle inhibitors p53/p21 suggesting that p53 is an important mediator of apoptosis and cell cycle arrest produced by TRF2 deficiency (Fig. 2). To address whether p53 deficiency could rescue TRF2-mediated proliferative and developmental defects *in vivo*, we generated mice simultaneously deficient for TRF2 and p53, *TRF2*^*Δ/Δ*^*-K5-Cre p53*^*−/−*^ mice. Similar to *TRF2*^*Δ/Δ*^*-K5-Cre* mice, *TRF2*^*Δ/Δ*^*-K5-Cre p53*^*−/−*^ double mutant were born at submendelian ratios and newborn mice died immediately after birth, indicating occurrence of embryonic lethality (Fig. 3C,D). No differences in neonate body weight were observed between *TRF2*^*Δ/Δ*^*-K5-Cre* and *TRF2*^*Δ/Δ*^*-K5-Cre p53*^*−/−*^ mice (Fig. 3E), suggesting that p53 deficiency does not rescue the mouse phenotype associated with TRF2 deficiency.

**Figure 4:**
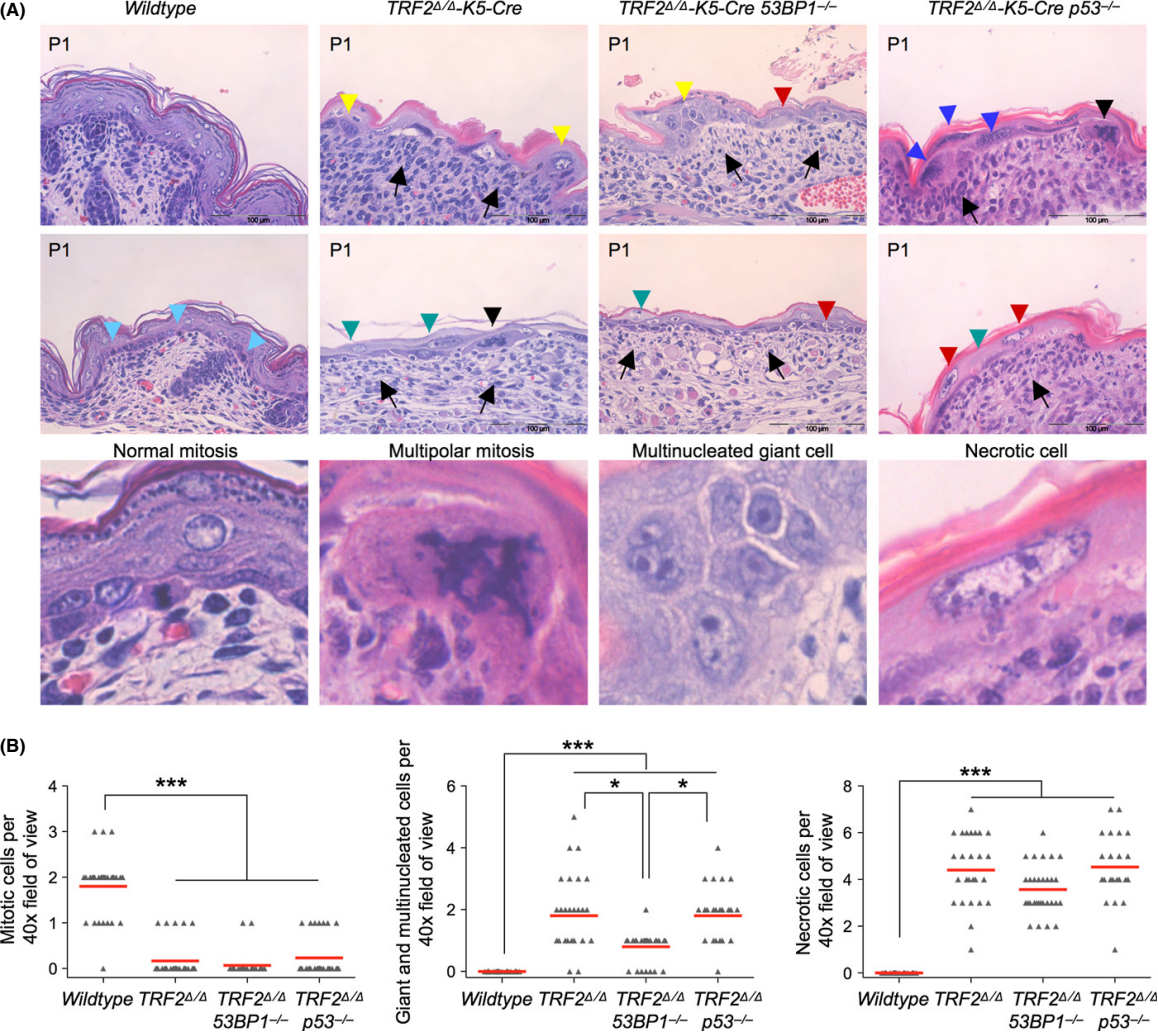
TRF2 deficient keratinocytes show aberrant mitotic figures, giant multinucleated cells, and necrotic cell features independently of 53BP1 and p53 status. (A) Histopathological findings of wild-type, *TRF2*^*∆/∆*^ *K5-Cre, TRF2*^*∆/∆*^ *K5-Cre 53BP1*^*−/−*^ and *TRF2*^*∆/∆*^ *K5-Cre p53*^*−/−*^ newborn epidermis. Black arrow, inflammatory cells in upper dermis; pale blue arrow head, normal mitosis; black arrow head, aberrant mitosis; yellow arrow head, multinucleated giant cells; blue arrow head, giant nuclei indicative of endoreduplication, red arrow head, necrotic cells with eosinophilic cytoplasm and euchromatic swollen nucleus; green arrow head, pale necrotic nuclei. In the lower panel, a larger magnification of a normal mitosis, a multipolar mitotic figure, a multinucleated giant cell, and a necrotic cell. (B) Quantification of mitotic cells, giant/multinucleated cells and necrotic cells observed in neonate skin of the indicated genotype. Of note, the scarce mitotic cells observed in TRF2-deleted skin presented a highly aberrant morphology. *P = 0.05; ***P < 0.001.

Histopathological analysis of *TRF2*^*Δ/Δ*^*-K5-Cre*, *TRF2*^*Δ/Δ*^*-K5-Cre 53BP1*^*−/−*^ and *TRF2*^*Δ/Δ*^*-K5-Cre p53*^*−/−*^ neonates revealed that the overall clinical picture was equally severe in the single knockout as in the compound mice under study (Fig 4). The wild-type neonatal skin clearly shows the basal layer, stratum granulosum, stratum corneum, and differentiated hair follicles. In contrast, the neonatal skin of *TRF2*^*Δ/Δ*^*-K5-Cre*, *TRF2*^*Δ/Δ*^*-K5-Cre 53BP1*^*−/−*^*,* and *TRF2*^*Δ/Δ*^*-K5-Cre p53*^*−/−*^ mice were extensively undeveloped, consisting of rare small regions that were largely necrotic (Fig 4A). These were highly dysplastic, with areas of hyperkeratosis and ulcerated parts. *TRF2*^*Δ/Δ*^*-K5-Cre 53BP1*^*−/−*^ and *TRF2*^*Δ/Δ*^*-K5-Cre p53*^*−/−*^ tongue, esophagus, and palate showed severe dysplasia similar to that formed in *TRF2*-null epithelia (data not shown). To address whether 53BP1- or p53-deficiency could be partially rescuing proliferative and stem cell defects observed during embryonic development of the *TRF2*^*Δ/Δ*^*-K5-Cre* single knockout mouse, we analyzed p63 expression and quantified the number of mitotic cells by phospho-histone 3 staining in E13.5 embryos (Fig. S3A,B). The p63 staining pattern in *TRF2*^*Δ/Δ*^*-K5-Cre*, *TRF2*^*Δ/Δ*^*-K5-Cre 53BP1*^*−/−*^*,* and *TRF2*^*Δ/Δ*^*-K5-Cre p53*^*−/−*^ embryos was similar and revealed a strong reduction in the number of basal cells as compared to wild-type embryos (Fig. S3A). No differences in the number of mitotic cells between the *TRF2*^*Δ/Δ*^*-K5-Cre* and *TRF2*^*Δ/Δ*^*-K5-Cre 53BP1*^*−/−*^ embryos were detected. However, at this stage, E13.5, p53 deficiency resulted in a bypass of TRF2-mediated mitotic arrest that was clearly not sufficient to prevent TRF2-mediated degenerative epithelial pathologies or survival (Fig. S3B). In fact, almost no mitotic cells in *TRF2*^*Δ/Δ*^*-K5-Cre*, *TRF2*^*Δ/Δ*^*-K5-Cre 53BP1*^*−/−*^*,* and *TRF2*^*Δ/Δ*^*-K5-Cre p53*^*−/−*^ neonates were detected as compared to wild-type neonates, which show 2 mitotic cells per visual field. Moreover, the scarce mitotic cells observed presented highly aberrant morphology (Black arrow heads in Fig 4A). Not a single normal mitotic figure was observed in TRF2-deficient epidermis regardless of 53BP1 and p53 status.

Histopathological examination of *TRF2*^*Δ/Δ*^*-K5-Cre*, *TRF2*^*Δ/Δ*^*-K5-Cre 53BP1*^*−/−*^*,* and *TRF2*^*Δ/Δ*^*-K5-Cre p53*^*−/−*^ skin revealed aberrant mitotic figures and giant multinucleated cells, which are hallmarks of mitotic catastrophe. Specifically, *TRF2*^*Δ/Δ*^*-K5-Cre, TRF2*^*Δ/Δ*^*-K5-Cre 53BP1*^*−/−*^*,* and *TRF2*^*Δ/Δ*^*-K5-Cre p53*^*−/−*^ neonates showed a significantly increased number of giant and multinucleated cells per visual field as compared to wild-type (Fig. 4B). Mitotic catastrophe has been delineated as a type of cell death that results from abnormal mitosis and is associated with premature chromosome condensation and the formation of large cells with multiple micronuclei (Vitale *et al*., 2011). Cellular composition of *TRF2*^*Δ/Δ*^*-K5-Cre p53*^*−/−*^ skin showed very few enormous cells, which presented giant nuclei and dense chromatin, a strong indication of the occurrence of massive endoreduplication (blue arrow heads in Fig. 4A). Of note, *TRF2*^*Δ/Δ*^*-K5-Cre* and *TRF2*^*Δ/Δ*^*-K5-Cre p53*^*−/−*^ present twofold increase in the number of giant and multinucleated cells than *TRF2*^*Δ/Δ*^*-K5-Cre 53BP1*^*−/−*^ indicating that 53BP1 deficiency partially rescues endoreduplication most likely due to less amount of fused chromosomes in the latter (Fig. 4B, see Fig. 6D,E). Morphologies characteristic of necrotic cells were observed at similar frequencies in *TRF2*^*Δ/Δ*^*-K5-Cre*, *TRF2*^*Δ/Δ*^*-K5-Cre 53BP1*^*−/−*^*,* and *TRF2*^*Δ/Δ*^*-K5-Cre p53*^*−/−*^ neonates, indicating that necrotic cell death is observed independently of either 53BP1 or p53 status (Fig. 4B). Necrotic cells present pale eosinophilic cytoplasm, swollen nucleus, euchromatic chromatin that condensates into irregular patches and increased cell volume that culminate in the breakdown of the plasma membrane (Fig 4). Of note, at this stage, cells with morphology characteristic of apoptotic cells were not observed (see Fig. 2E).

### p53 mediates p21 induction and apoptosis upon *TRF2* deletion during embryonic development

**Figure 5:**
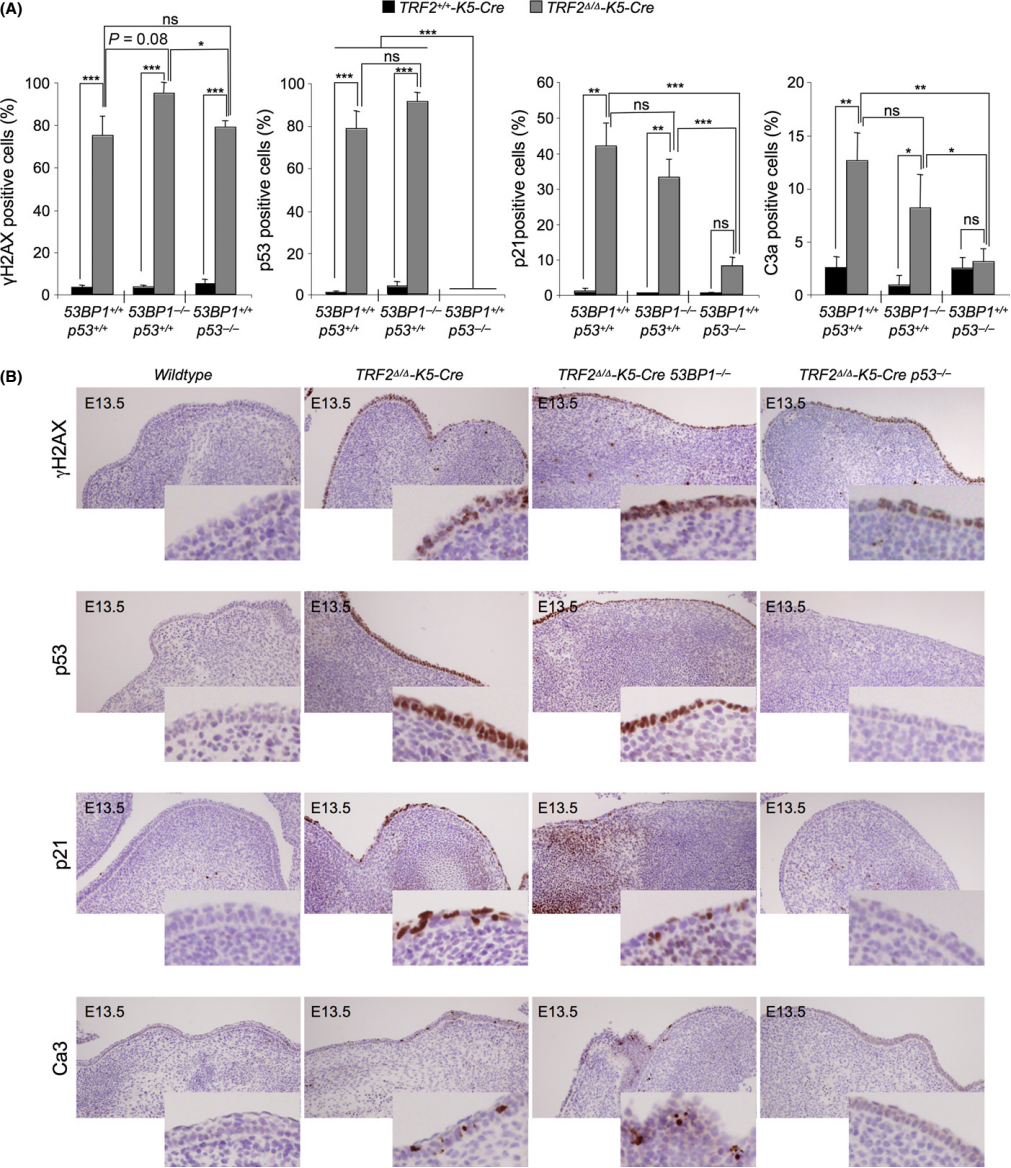
p53 mediates p21 induction and apoptosis upon *TRF2* deletion during embryonic development. (A) Percentage of epidermal cells showing γH2AX foci, p53 positive cells, p21 positive cells, and apoptotic C3a positive cells in wild-type, *TRF2*^*∆/∆*^ *K5-Cre, TRF2*^*∆/∆*^ *K5-Cre 53BP1*^*−/−*^ and *TRF2*^*∆/∆*^ *K5-Cre p53*^*−/−*^ E13.5 embryos. (B) Representative images of γH2AX, p53, p21, and Ca3 immunohistochemistry staining in E13.5 embryos of the indicated genotypes. Amplified images are shown in the insets. *n* = 2–4 embryos analyzed for genotype. Error bars indicate standard error. Student’s *t*-test was used for statistical analysis. *P = 0.05; **P < 0.01; ***P < 0.001.

**Figure 6:**
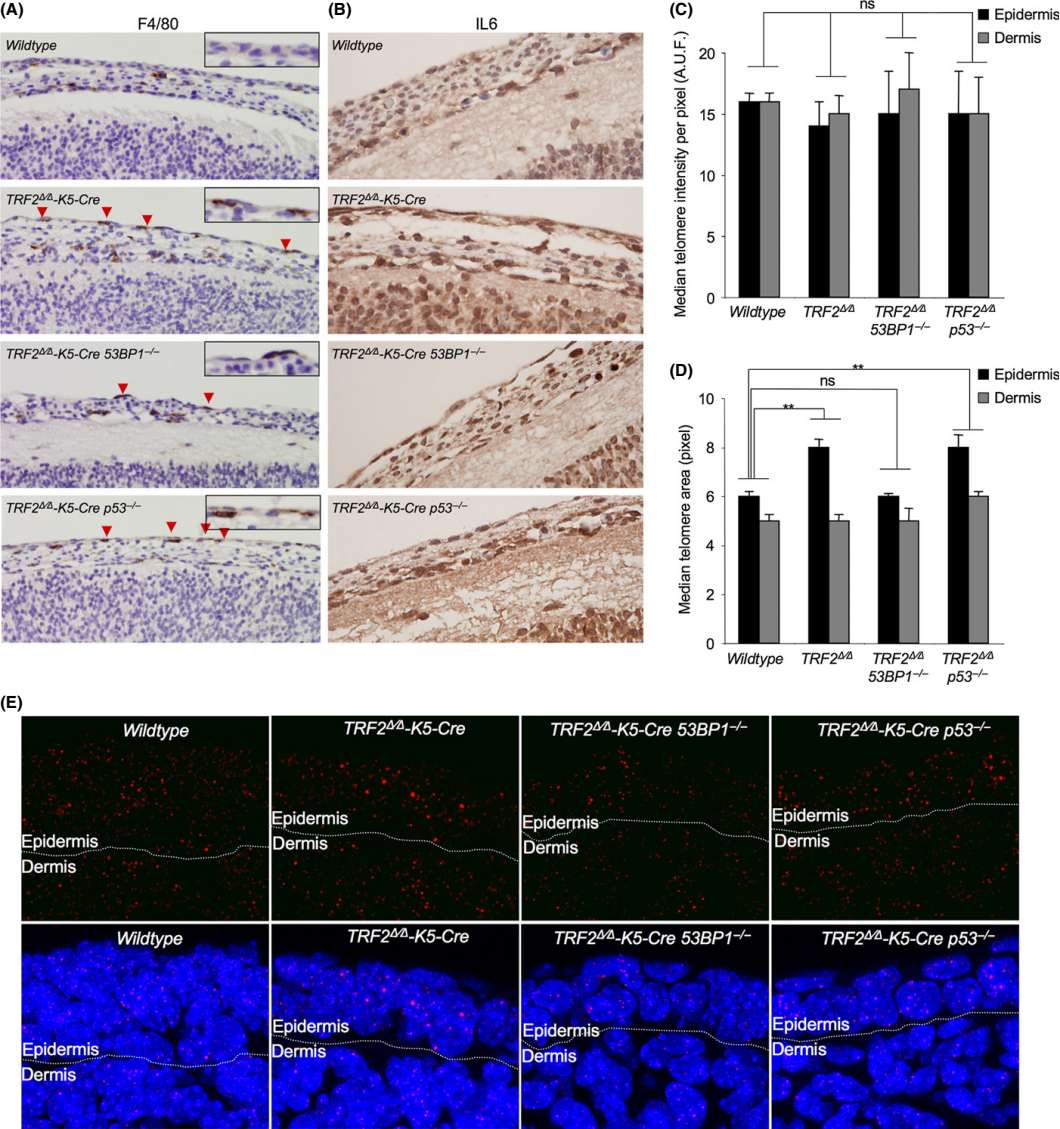
TRF2 deficiency leads to inflammation and to chromosomal end-to end fusions in stratified epithelia. (A, B) Representative images of F4/80 (A) and interleukin 6 (B) immunohistochemistry staining in wild-type, *TRF2*^*∆/∆*^ *K5-Cre, TRF2*^*∆/∆*^ *K5-Cre 53BP1*^*−/−*^ and *TRF2*^*∆/∆*^ *K5-Cre p53*^*−/−*^ E13.5 embryos. Note the increased IL6 levels and the presence of F4/80 positive cells in the epidermal layer of embryos deficient for TRF2 independently of 53BP1 and p53 status. (C,D) Median of the distribution of individual spot fluorescence per pixel (C) and median of the distribution of individual spot area (D) determined by quantitative telomere FISH (Q-FISH) in epidermal layer (black bars) and in subadjacent nonepidermal tissues (gray bars) of E13.5 embryo sections of the indicated genotypes. Note the increase in the median value of spot area distribution in *TRF2*^*∆/∆*^ *K5-Cre* and in *TRF2*^*∆/∆*^ *K5-Cre p53*^*−/−*^ as compared to wild-type and *TRF2*^*∆/∆*^ *K5-Cre 53BP1*^*−/−*^ in the epidermal tissue, while no differences are observed among genotypes in the subadjacent nonepidermal tissues. *n* = 2–5 embryos per genotype. Error bars indicate standard error. Statistical significance was determined by two-tailed Student’s t-test taking the mean values of individual embryos. A.U.F., arbitrary units of fluorescence. (E) Representative images of telomere Q-fish images on skin sections in E13.5 embryos of the indicated genotypes where DAPI nuclear staining is shown in blue and telomere signal in red. **P < 0.01.

To address whether either 53BP1 or p53 deficiency could have an impact on the DNA damage level, on the induction of p53/p21 as well as in apoptotic levels during fetus development, we performed a comparative study among *TRF2*^*Δ/Δ*^*-K5-Cre 53BP1*^*+/+*^ *p53*^*+/+*^, *TRF2*^*Δ/Δ*^*-K5-Cre 53BP1*^*−/−*^ *p53*^*+/+*^*, TRF2*^*Δ/Δ*^*-K5-Cre 53BP1*^*+/+*^*p53*^*−/−*^*,* and their correspondent wild-type and single knock-out controls in embryos at E.13.5, two days after Cre expression. Analysis of γH2AX positive cells in stratified epithelia did not reveal significant differences between *TRF2*^*Δ/Δ*^*-K5-Cre p53*^*+/+*^ and *TRF2*^*Δ/Δ*^*-K5-Cre p53*^*−/−*^ embryos, being approximately 75% of the cells γH2AX positive (Fig. 5A,B). However, 100% of the cells in *TRF2*^*Δ/Δ*^*-K5-Cre 53BP1*^*−/−*^ E.13.5 embryos were positive for γH2AX. This increase could be explained by the abolishment of chromosome end-to-end fusions that temporarily leads to higher number of exposed damaged telomeres as compared to cells proficient in the C-NHEJ (see Fig. 6). The number of p53 positive cells in *TRF2*^*Δ/Δ*^*-K5-Cre 53BP1*^*−/−*^ embryos was also somewhat higher as compared to *TRF2*^*Δ/Δ*^*-K5-Cre 53BP1*^*+/+*^ E.13.5 embryos although this difference did not reach significant values (Fig. 5). As expected, no p53 signal was detected in *p53*^*−/−*^ samples. The number of p21 positive cells was similarly increased in *TRF2*^*Δ/Δ*^*-K5-Cre 53BP1*^*+/+*^ *p53*^*+/+*^ and *TRF2*^*Δ/Δ*^*-K5-Cre 53BP1*^*−/−*^ *p53*^*+/+*^ embryos, suggesting that is independent of the 53BP1 expression. However, the percentage of p21-positive cells was significantly decreased in *TRF2*^*Δ/Δ*^*-K5-Cre 53BP1*^*+/+*^ *p53*^*−/−*^ epithelia as compared to *TRF2*^*Δ/Δ*^*-K5-Cre 53BP1*^*+/+*^ *p53*^*+/+*^ epithelia, in agreement with the fact that p21 activation is mediated by p53 (Fig. 5).

Apoptotic levels were similar between *TRF2*^*Δ/Δ*^*-K5-Cre 53BP1*^*+/+*^ *p53*^*+/+*^ and *TRF2*^*Δ/Δ*^*-K5-Cre 53BP1*^*−/−*^ *p53*^*+/+*^ embryos, reaching values over 10% of the total epidermal cells (Fig. 5), suggesting that the apoptotic response is not triggered by the presence of chromosome fusions. In contrast, the number of caspase-3 (Ca3) positive cells was not further increased in *TRF2*^*Δ/Δ*^*-K5-Cre 53BP1*^*+/+*^ *p53*^*−/−*^ compared with *TRF2*^*+/+*^ control embryos. These results indicate that the increase in the Ca3 positive cells observed upon TRF2 removal is p53-mediated. Similar values than with Ca3 staining were obtained by TUNEL assay indicating that the observed apoptosis was Ca3-mediated (Figure S3C). The observation that abolition of p53/p21 response as well as apoptosis by p53 deficiency did not rescue developmental defects and survival of the *TRF2*^*Δ/Δ*^*-K5-Cre p53*^*−/−*^ mice indicates that these two processes, cell cycle arrest and programmed cell death induced by TRF2 deletion and mediated by p53, are not the major cellular responses responsible for the severe skin atrophy and morbidity of TRF2-deleted mice. Rather, it appears that extensive necrosis (see Fig. 4) and inflammation are the causes of the severity of the phenotype. Indeed, the presence of macrophage infiltrates became apparent by immunohistochemistry analysis of F4/80 positive cells, a marker of mature macrophages, in epidermal layer already at E13.5 embryos lacking TRF2 (Fig. 6A). Moreover, TRF2-deficient E13.5 embryos independently of 53BP1 and p53 status showed increased interleukin 6 (IL6) levels as compared to wild-type embryos, indicative of an inflammatory process triggered by tissue damage (Fig. 6B).

### TRF2 deficiency does not impact on telomere length homeostasis

To address whether TRF2 deletion in stratified epithelia could lead to defects in telomere length homeostasis, as well as to confirm that 53BP1 deficiency also abolishes end-to-end fusions *in vivo*, we performed quantitative telomere FISH (QFISH) analysis on wild-type, *TRF2*^*Δ/Δ*^ *K5-Cre, TRF2*^*Δ/Δ*^ *K5-Cre 53BP1*^*−/−*^*,* and *TRF2*^*Δ/Δ*^ *K5-Cre p53*^*−/−*^ E13.5 embryo sections. We quantified the mean intensity fluorescence per area of the spot and the area of each individual spot in both the epidermal layer as well as in the subadjacent nonepidermal tissue of the embryos (Fig. 6C–E and Fig. S4). We did not find significant differences in the median telomere fluorescence per pixel neither when comparing the epidermis with the dermis within a given genotype nor among the different genotypes analyzed, indicating that *TRF2* deletion specifically in the epidermis does not impact on telomere length (Fig. 6C and Fig. S4). Interestingly, when we calculated the median telomere area, we found significantly higher telomere area in *TRF2*^*Δ/Δ*^ *K5-Cre* and *TRF2*^*Δ/Δ*^ *K5-Cre p53*^*−/−*^ epidermis compared with wild-type and with *TRF2*^*Δ/Δ*^ *K5-Cre 53BP1*^*−/−*^ epidermis (Fig. 6D,E and Fig. S4). These differences, however, were not observed in the dermis where *TRF2* is not deleted, indicating that are the consequence of *TRF2* deletion. These results are compatible with a higher portion of telomeric association/fusions and therefore higher telomeric area in TRF2-deficient epidermis, which are rescued in the absence of 53BP1 but not in the absence of p53 (Fig. 6D,E and Fig. S4). These findings are in agreement with the fact that 53BP1 but not p53 is involved in the C-NHEJ pathway.

## Discussion

The telomere-binding protein TRF2 has been extensively studied *in vitro*. In particular, *In vitro* studies have shown that TRF2 has a crucial role in end protection through the suppression of ATM activation and inhibiting the NHEJ pathway and thereby preventing end-to-end fusions (van Steensel *et al*., 1998; Karlseder *et al*., 2004; Celli & de Lange, 2005; Denchi & de Lange, 2007; Okamoto *et al*., 2013). In contrast, there are very few reports on the function of TRF2 in the organism. In particular, while whole body deletion of *TRF2* causes fully penetrant embryonic lethality (Celli & de Lange, 2005), *TRF2* deletion in the liver is dispensable for liver regeneration and mouse survival (Lazzerini Denchi *et al*., 2006). In this work, we have studied the impact of *TRF2* conditional deletion in stratified epithelia during embryonic development. Previous work from our group has shown the impact of deleting other members of the shelterin complex in the skin (i.e., TRF1, TPP1, and RAP1) (Martinez *et al*., 2009, 2010; Tejera *et al*., 2010). Therefore, it would be of interest to determine the relative importance of the different shelterin components in tissue homeostasis. We show that embryonic development of *TRF2*^*Δ/Δ*^*-K5-Cre* epidermis is fully blocked from E11.5 at the time when K5-Cre expression commences. *TRF2* deletion provokes a dramatic DNA damage burden and an immediate DNA damage response as indicated by the increase in γH2AX, p53, and p21 positive cells in the epidermal layer of the skin that is coincidental with a progressive induction of apoptosis until E16.5. Indeed, epidermis of E16.5 embryos displayed only one keratinocytes layer and extensive necrosis as well as complete absence of early hair primordia. Skin of E18.5 embryos is composed of rare small areas of epidermis, which were completely necrotic and keratinocytes presented marked cytological atypia with disturbed polarity of the cells and extremely large nuclei. The clinical picture worsens with the progress of embryonic development, becoming incompatible with life in newborns. This is in agreement with the observed partial embryonic lethality and immediate death after birth of the *TRF2*^*Δ/Δ*^*-K5-Cre* newborns. In comparison with other available conditional knock-out mice in stratified epithelia lacking shelterin components, that is, *TRF1*^*Δ/Δ*^*-K5-Cre, TPP1*^*Δ/Δ*^*-K5-Cre,* and *RAP1*^*Δ/Δ*^*-K5-Cre*, the *TRF2*^*Δ/Δ*^*-K5-Cre* mouse is the only one that shows partial embryonic lethality (Martinez *et al*., 2009, 2010; Tejera *et al*., 2010). Indeed, although both *TRF1*^*Δ/Δ*^*-K5-Cre* and *TPP1*^*Δ/Δ*^*-K5-Cre* mice show perinatal lethality, some neonates reached day 6 and day 9 postpartum, respectively, while no impact on survival was observed in the *RAP1*^*Δ/Δ*^*-K5-Cre* as compared to their wild-type counterparts (Martinez *et al*., 2009, 2010; Tejera *et al*., 2010). In addition, morphological defects of the *TRF2*^*Δ/Δ*^*-K5-Cre* epidermis were more severe as compared to those showed by the *TRF1*^*Δ/Δ*^*-K5-Cre* and *TPP1*^*Δ/Δ*^*-K5-Cre* neonates (Martinez *et al*., 2009; Tejera *et al*., 2010). In contrast to the rescue of degenerative pathologies observed by p53 deficiency combined with *TRF1* and *TPP1* deletion, p53 abrogation did not bypass developmental defects or perinatal lethality associated with TRF2 loss (Martinez *et al*., 2009; Tejera *et al*., 2010). Interestingly, although the percentage of both p21 and apoptotic cells in the *TRF2*^*Δ/Δ*^ *K5-Cre p53*^*−/−*^ E13.5 embryos remained similar to that of *TRF2*^*+/+*^ wild-type embryos, this was no sufficient to recover either epidermal development or embryonic viability.

Based on histological findings of TRF2-deficient epithelia revealing the presence of aberrant mitosis, giant multinucleated cells and morphologies characteristic of necrotic cells as well as on the observation that abolition of senescence and apoptosis by combined p53-deficiency did not rescue TRF2-associated phenotypes, we propose that mitotic catastrophe and necrosis are the cause of complete disappearance of the epidermal basal layer already from E16.5 onwards. Mitotic catastrophe has been extensively used as a term to indicate cell death resulting from aberrant mitosis (Kroemer *et al*., 2009). Some studies show that mitotic catastrophe can be followed by apoptosis and is therefore a matter of debate whether mitotic catastrophe is a specific death process or just functions as a trigger for other cell death mechanisms (Castedo *et al*., 2004). The actual view defines mitotic catastrophe as a mechanism that senses mitotic failure and responds to it by driving the cell to an irreversible fate, be it apoptosis, necrosis or senescence-mediated elimination of mitosis deficient, genomically unstable cells. (Vitale *et al*., 2011). Unfortunately, given the impossibility to cell culture TRF2-deficient keratinocytes, we cannot provide a molecular probe to ultimately demonstrate necrosis as the mechanism by which cell death is taking place upon TRF2 loss in skin (Li *et al*., 2012). However, the fact that TRF2-driven apoptosis and senescence were abolished in *TRF2*^*Δ/Δ*^ *K5-Cre p53*^*−/−*^ embryos without impacting on survival supports the statement that disappearance of epidermal layer in TRF2 deficient mice is mainly necrosis-mediated.

The current view is that necrotic cell death is not the result of one well-defined signaling cascade but rather the consequence of crosstalk between several signaling pathways (Festjens *et al*., 2006; Kung *et al*., 2013). Apoptosis occurs without and sometimes even with the active sequestration of danger-associated molecular pattern. Apoptotic corpses can suppress the transcription of proinflammatory cytokine genes, promote the secretion of anti-inflammatory cytokines by phagocytes and cause antigen-presenting cells to present dead-cell-antigen leading to immunological tolerance. In contrast, necrotically dying cells initiate proinflamatory signaling cascade by actively releasing inflammatory cytokines and by spilling their contents to the environment when they lyse to attract inflammatory effectors and in particular macrophages (Zitvogel *et al*., 2010). Indeed, the presence of macrophage infiltrates and increased levels of IL6 became apparent in epidermal layer already at E13.5 embryos lacking TRF2 indicating commence of tissue inflammation. TRF2 deletion also releases RAP1 from telomeres. RAP1 has been shown to act as a transcriptional regulator (Martinez *et al*., 2010). It is therefore tempting to speculate that release of RAP1 from telomeres upon TRF2 deletion might be upregulating pronecrosis genes. Confirmation of this hypothesis warrants further studies. Interestingly, it has been shown that extensive DNA damage induces necrotic cell death in a Poly(ADP-ribose) polymerase (PARP) dependent manner. PARP is a nuclear enzyme activated by DNA strand breaks that participates in DNA repair. Overactivation of PARP after extensive DNA damage leads to necrotic cell death by depletion of β-nicotinamide adenine dinucleotide and ATP. In fact, PARP-deficient mouse fibroblasts are protected from necrotic cell death and ATP depletion but not from apoptotic death (Ha & Snyder, 1999). It would be interesting to test whether PARP-deficiency may rescue epidermal developmental defect associated with TRF2-loss. [Correction added on 30 June 2014, after first online publication: the abbreviation of Poly(ADP-ribose) polymerase was corrected from PPAR to PARP.]

Abolishment of end-to-end chromosomal fusions by 53BP1 deficiency neither rescued nor aggravated TRF2-mediated developmental and molecular skin defects. This is in marked contrast to *TRF1*^*Δ/Δ*^*-K5-Cre 53BP1*^*−/−*^ mice that showed a clear aggravation of the skin phenotypes as compared to *TRF1*^*Δ/Δ*^*-K5-Cre* single mutant, which was associated with an earlier onset of DNA damage and DDR activation during embryonic development (Martinez *et al*., 2012). In fact, 2 days after Cre expression in E13.5 *TRF2*^*Δ/Δ*^*-K5-Cre 53BP1*^*−/−*^ embryos, 100% of the cells were positive for γH2AX indicating that chromosomal fusions are not the cause of embryonic death but rather the high DNA damage burden occurring upon TRF2 depletion. The differences obtained between the *TRF1*^*Δ/Δ*^*-K5-Cre 53BP1*^*−/−*^ and the *TRF2*^*Δ/Δ*^*-K5-Cre 53BP1*^*−/−*^ mice could be due to the more immediate and stronger DNA damage inflicted upon TRF2 depletion as compared to TRF1. Our results show that *in vivo* TRF2 abrogation in a highly proliferative tissue such as skin leads to the most severe deleterious effect as compared to TRF1, TPP1, and RAP1 loss (Martinez *et al*., 2009, 2010; Tejera *et al*., 2010).

Despite the deleterious molecular consequences of TRF2 loss, conditional deletion of *TRF2* in the liver did not impact on liver regeneration or mouse viability (Lazzerini Denchi *et al*., 2006). The very distinct outcomes obtained from deleting *TRF2* in the liver or in the epidermis must be due to the different nature of the tissue. In noncycling hepatocytes, TRF2-mediated telomere dysfunction did not induce p53 or cell death and liver function was unaltered (Lazzerini Denchi *et al*., 2006). In contrast, in highly proliferative tissues such as skin, *TRF2* deletion induces a rapid DDR that leads to inhibition of cell proliferation, cell death, and ultimately to tissular necrosis.

## Experimental procedures

### Generation of mice

Homozygous *TRF2*^*flox/flox*^ mice were purchased from The Jackson Laboratories (www.Jax.org) (Celli & de Lange, 2005) and crossed with transgenic mice expressing the Cre recombinase under the control of the keratin 5 promoter (Tarutani *et al*., 1997). Heterozygous *TRF2*^*+/Δ*^*-K5-Cre* were crossed either to *TRF2*^*flox/flox*^ or *TRF2*^*+/flox*^ to generate *TRF2*^*Δ/Δ*^*-K5-Cre* mice. To generate *TRF2*^*Δ/Δ*^*K5-Cre 53BP1*^*−/−*^mice, *TRF2*^*+/∆*^*K5-Cre 53BP1*^*+/+*^ mice were first crossed to *TRF2*^*+/+*^*53BP1*^*−/−*^ mice (Ward *et al*., 2003). The crosses between double heterozygote *TRF2*^*+/flox*^ *53BP1*^*+/-*^and *TRF2*^*+/Δ*^*K5-Cre 53BP1*^*+/-*^ mice generated double homozygote *TRF2*^*Δ/Δ*^*K5-Cre 53BP1*^*−/−*^mice. Similarly, to generate *TRF2*^*Δ/Δ*^*K5-Cre p53*^*−/−*^ mice, *TRF2*^*+/+*^ *p53*^*−/−*^ mice were first crossed to *TRF2*^*+/∆*^*K5-Cre p53*^*+/+*^ mice. The crosses between double heterozygote *TRF2*^*+/flox*^ *p53*^*+/-*^and *TRF2*^*+/Δ*^*K5-Cre p53*^*+/-*^ mice generated double homozygote *TRF2*^*Δ/Δ*^*K5-Cre p53*^*−/−*^mice. All mice were generated and maintained at the Spanish National Cancer Center under specific pathogen-free conditions in accordance with the recommendation of the Federation of European Laboratory Animal Science Associations.

### Histopathological analysis, immunohistochemistry, and immunofluorescence staining techniques

Skin samples and other mouse tissues were fixed in 10% buffered formalin, dehydrated, and embedded in paraffin. For histopathological analysis, 4-μm sections were deparaffinated and stained with hematoxylin and eosin according to standard procedures. A minimum of five mice or embryos per genotype were histopathologically analyzed in back skin sections. A blind subjective image analysis to estimate the keratinocyte layers was performed blindly. Multinucleated, giant cells and aberrant mitosis figures were counted on a minimum of ten randomly selected 40× high-power fields per animal. Immunohistochemistry was performed on deparaffinated skin sections processed with 10 mm sodium citrate (pH 6.5) cooked under pressure for 2 min. Slides were washed in water, then in Buffer TBS Tween20 0.5%, blocked with peroxidase, washed with TBS Tween20 0.5% again, and blocked with fetal bovine serum followed by another wash. The slides were incubated with the primary antibodies: rabbit polyclonal to p53 (CM5, Novocastra), goat polyclonal to p21 (C-19-G, Santa Cruz), mouse monoclonal to phospho-histone H2AX (ser139), rabbit polyclonal to SOX9 (Millipore), mouse monoclonal to p63 (p63-4A4, Neo Markers), rabbit polyclonal to cytokeratine 14 (AF64, Covance), rabbit polyclonal to cytokeratine 10 (Covance), rabbit polyclonal to caspase-3 active (R&D systems), rabbit polyclonal to cytokeratine 6 (Covance), rabbit polyclonal to loricrine (Covance), rabbit polyclonal to phospho-histone 3 H3 (ser10) (Millipore), and rabbit polyclonal to interleukin 6 (ABCAM). Slides were then incubated with secondary antibodies conjugated with peroxidase from DAKO. For signal development, DAB (DAKO) was used as a substrate. Sections were lightly counterstained with hematoxylin and analyzed by light microscopy. Telomere-induced foci (TIFs) were detected using a rabbit polyclonal to TRF1 (homemade) and a mouse monoclonal antibody raised against phospho-histone2 H2AX-Ser 139 (Millipore). Centrosome and microtubules were detected using a rabbit polyclonal to pericentrina (Covance) and a mouse monoclonal to α-tubulin (abcam). DeadEnd Fluorometric TUNEL System was used to assay apoptotic cells according to the manufacturer’s instructions (Promega).

### Quantitative immuno fish

Quantitative telomere fluorescence *in situ* hybridization (Q-FISH) directly on embryo sections was performed as previously described (Zijlmans *et al*., 1997; Martinez *et al*., 2013). Confocal microscopy was performed at room temperature with a laser-scanning microscope (TSC SP5) using a Plan Apo 63Å-1.40 NA oil immersion objective (HCX). Maximal projection of z-stack images generated using advanced fluorescence software (LAS) was analyzed with Definiens XD software package. The DAPI images were used to detect telomeric signals inside each nuclei.

### Statistical analysis

A Student’s t-test was used to calculate the statistical significance of the observed differences in telomere fluorescence, γH2AX foci, 53BP1 foci, body weight, γH2AX expression, p53 expression, p21 expression, and caspase-3 expression. A chi-square test was used to calculate statistical significance in Mendelian ratios.

## Supporting Information

**Fig. S1** Aberrant expression pattern of differentiation markers in *TRF2*-null epidermis. **Fig. S2** TRF2 deficiency leads to severe epidermal stem cell defects. **Fig. S3** 53BP1 and p53 deficiencies do not rescue TRF2-associated proliferative defects. **Fig. S4** TRF2 deficiency does not impact in telomere length homeostasis in stratified epithelia.
