# Supplementary figures and images for: Essential role for the TRF2 telomere protein in adult skin homeostasis
Source: Aging Cell. 2014 Apr 14;13(4):656–68. doi: 10.1111/acel.12221 (PMC4326939; doi:10.1111/acel.12221)

Supplementary Figure 1

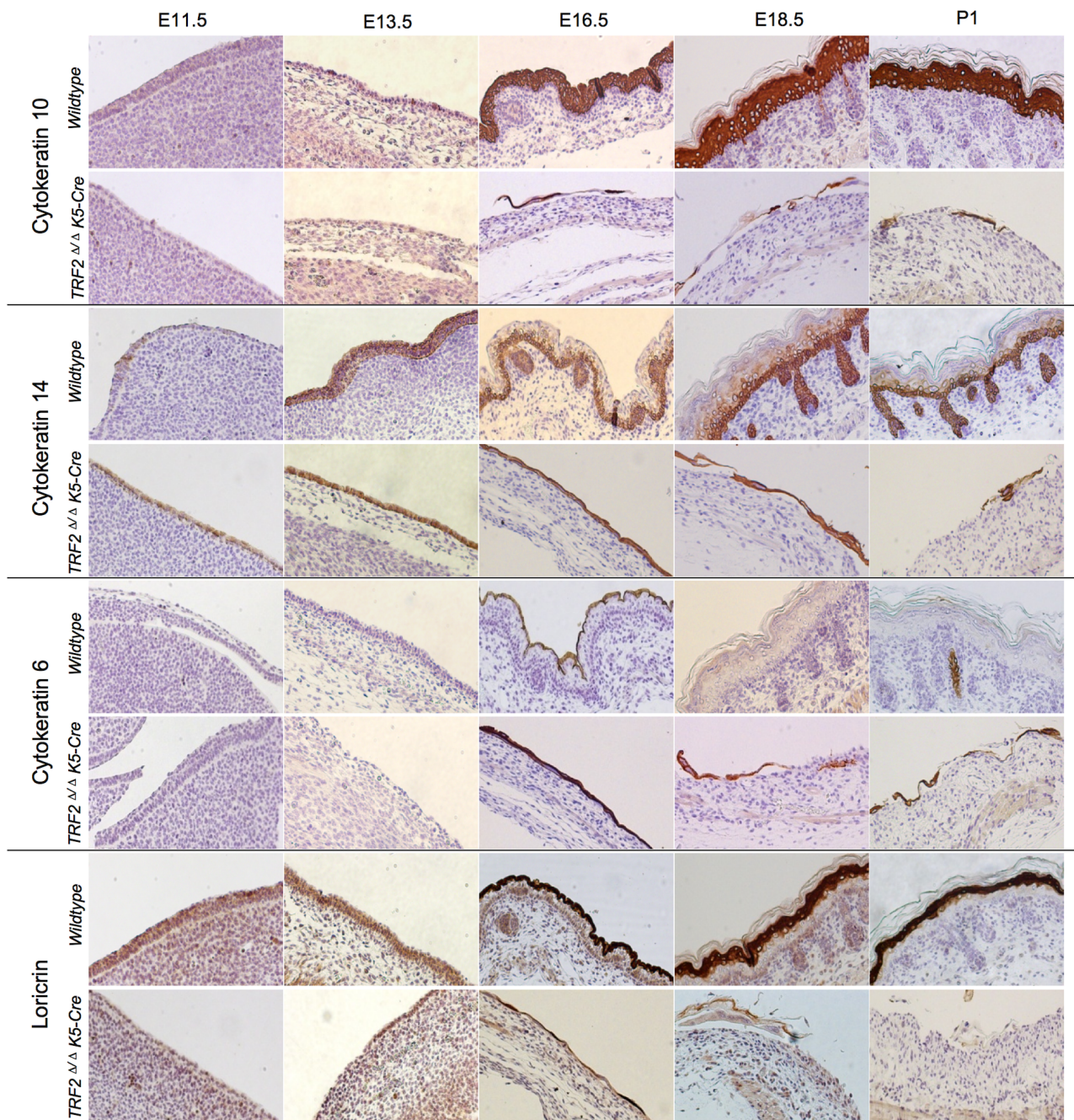

Supplementary Figure 2

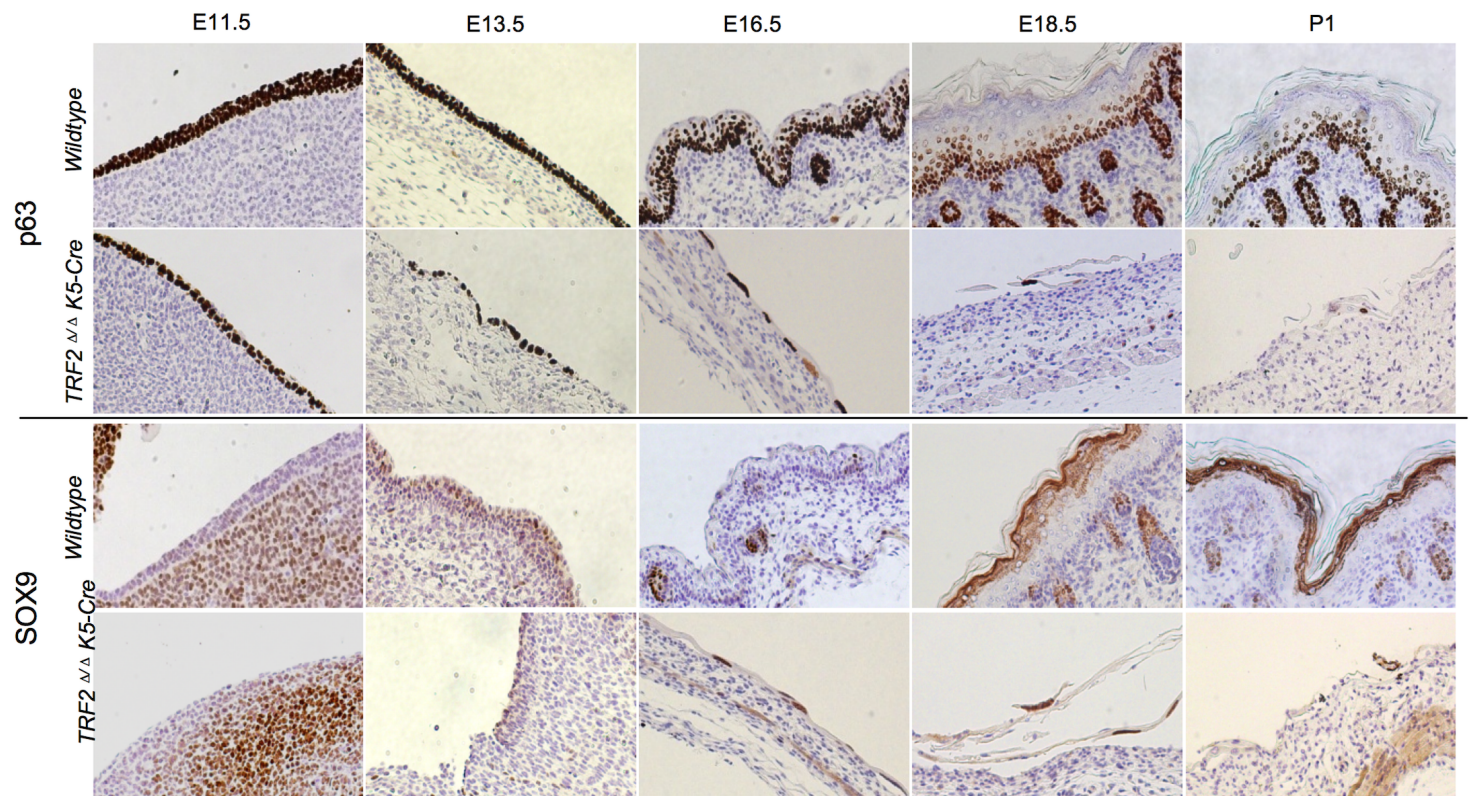

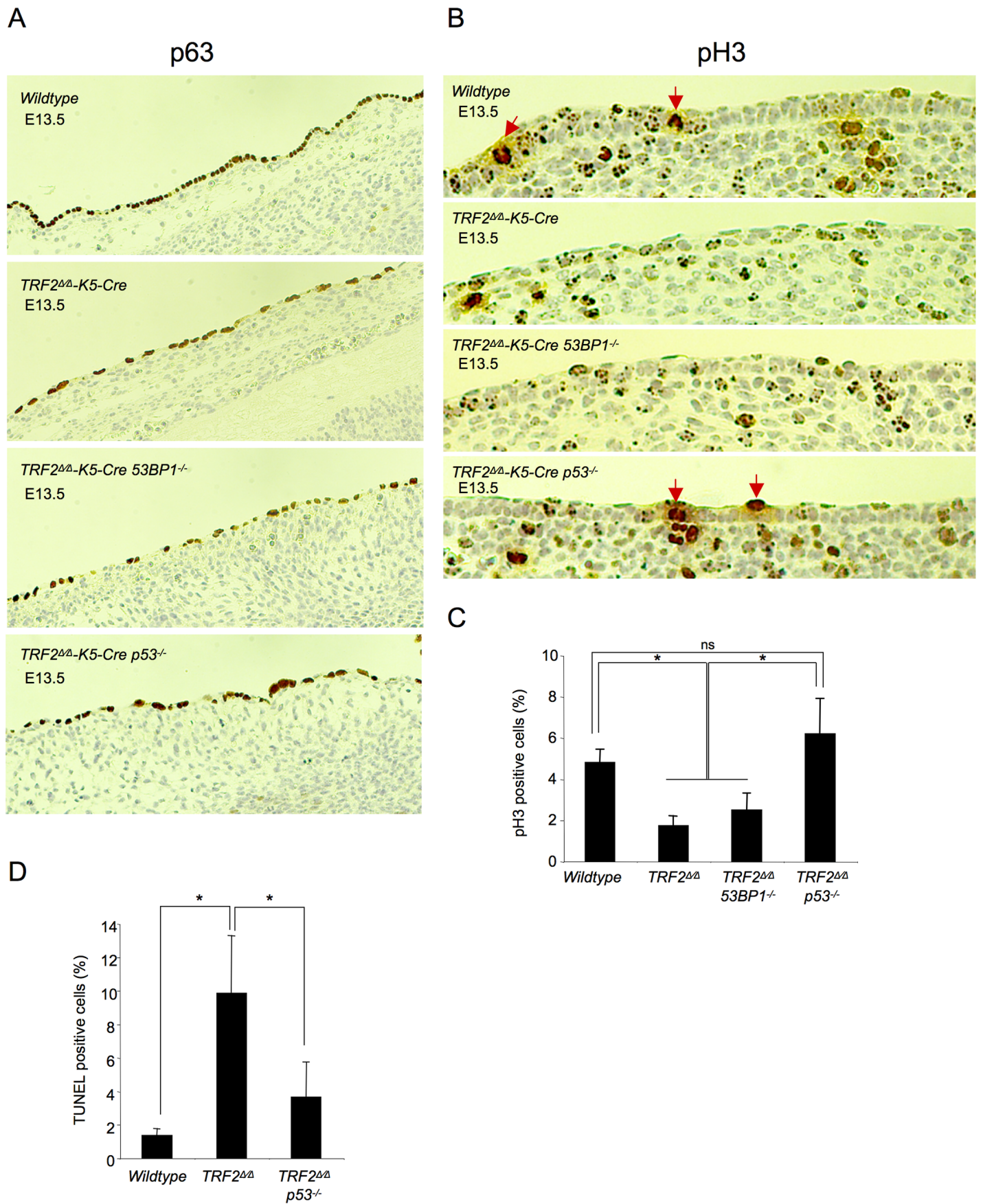

A

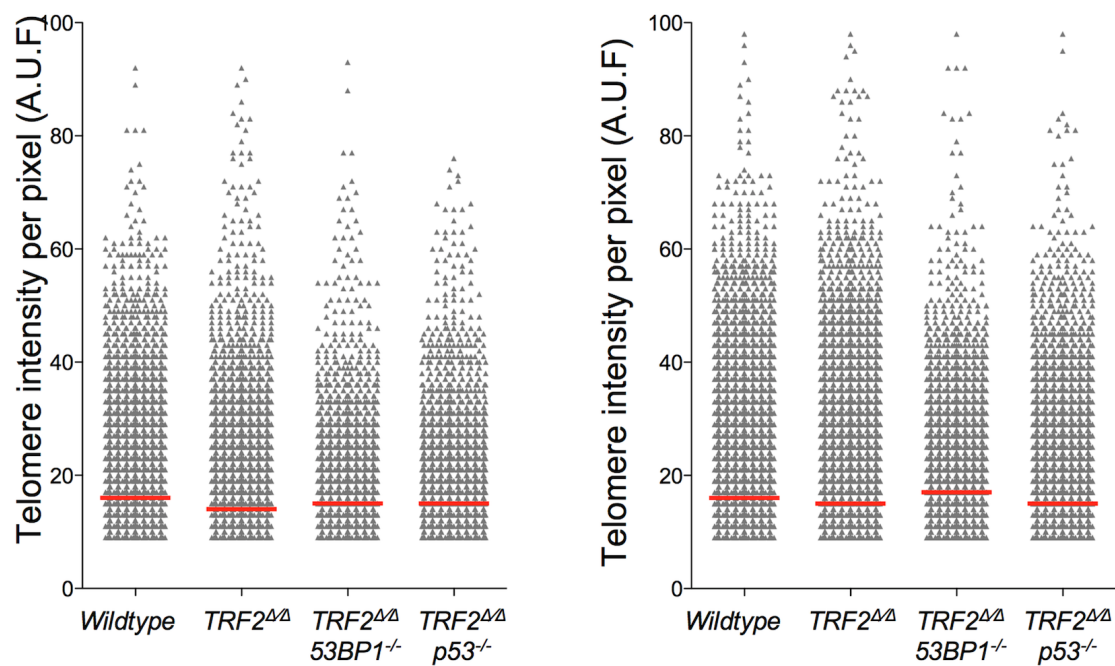

B

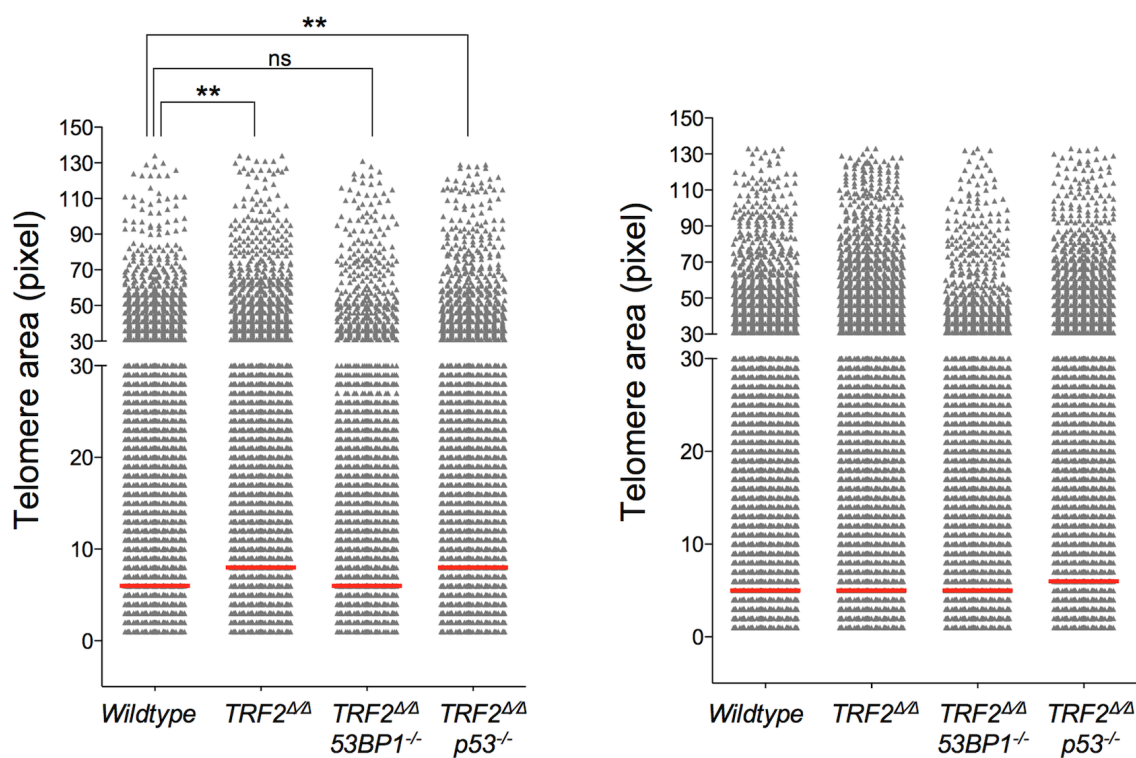

Supplement: Supplementary file 1 — Fig. S1 Aberrant expression pattern of differentiation markers in TRF2-null epidermis. Fig. S2 TRF2 deficiency leads to severe epidermal stem cell defects. Fig. S3 53BP1 and p53 deficiencies do not rescue TRF2-associated proliferative defects. Fig. S4 TRF2 deficiency does not impact in telomere length homeostasis in stratified epithelia. [file acel0013-0656-sd1.pdf]
